# Efficacy and safety of ferric citrate hydrate compared with sodium ferrous citrate in Japanese patients with iron deficiency anemia: a randomized, double-blind, phase 3 non-inferiority study

**DOI:** 10.1007/s12185-021-03123-9

**Published:** 2021-03-15

**Authors:** Norio Komatsu, Kojo Arita, Hironori Mitsui, Takanori Nemoto, Koji Hanaki

**Affiliations:** 1 Department of Hematology, Juntendo University School of Medicine, 2-1-1 Hongo, Bunkyo-ku, Tokyo, 113-8421 Japan; 2 Pharmaceutical Division, Japan Tobacco Inc., 3-4-1 Nihonbashi-Honcho, Chuo-ku, Tokyo, 103-0023 Japan

**Keywords:** Ferric citrate hydrate, Iron deficiency anemia, Oral iron preparation, Japan, Gastrointestinal side effects

## Abstract

Oral iron preparations are used as first-line treatment for iron deficiency anemia (IDA), but their gastrointestinal side effects prevent patients from appropriate adherence. We recently conducted a randomized, double-blind, phase 3 non-inferiority study to evaluate the efficacy and safety of two dosages of ferric citrate hydrate (FC) compared with sodium ferrous citrate (SF) in patients with IDA. FC at both 500 and 1000 mg/day was non-inferior to SF at 100 mg/day in terms of the change in the hemoglobin concentration at Week 7 from baseline. Logistic regression analysis suggested that the cumulative proportion of patients who achieved the target hemoglobin concentration (≥ 13.0 g/dL in male patients and ≥ 12.0 g/dL in female patients) at Week 7 was highest among those treated with FC at 1000 mg/day, followed by SF at 100 mg/day and FC at 500 mg/day. Both dosages of FC were well tolerated in patients with IDA. The incidences of nausea and vomiting were significantly lower in the FC treatment groups than in the SF group. In conclusion, FC has potential to be an oral iron preparation with sufficient efficacy for the treatment of IDA and a lower risk of nausea and vomiting.

**Supplementary Information:**

The online version contains supplementary material available at 10.1007/s12185-021-03123-9.

## Introduction

Iron deficiency anemia (IDA) is the most common type of anemia and is caused by the depletion of body iron [1–3], which is an essential component of heme and hemoglobin (Hb). Because intestinal iron absorption is controlled by the total amount of body iron and there are no passive mechanisms by which iron is excreted from the body, systemic iron homeostasis is strictly regulated [4]. Dietary intake of iron is considered adequate to replenish the loss of iron by sweating, urination, or desquamation of the intestinal epithelium. However, the low iron stores associated with chronic bleeding or reduction of iron intake lead to a decline in the whole blood Hb concentration.

Oral iron preparations are recommended as first-line treatment of IDA; intravenous iron preparations are only used in patients who have oral iron intolerance or a condition in which oral iron preparations are inappropriate. For example, it is suitable for patients who have difficulties in adherence due to gastrointestinal side effects by the exiting oral iron preparations or who have massive bleeding to treat with intravenous iron preparations. The oral iron preparations currently available in Japan have sufficient efficacy for the treatment of IDA, but gastrointestinal side effects such as nausea or vomiting appear in up to 30% of patients taking oral iron, which decreases patients’ adherence to appropriate treatment [5, 6]. Thus, a novel oral iron preparation with sufficient therapeutic efficacy and favorable gastrointestinal tolerance is still needed.

Each ferric citrate hydrate (FC) 250-mg tablet contains approximately 60 mg of elemental ferric iron. FC (Riona^®^, Japan Tobacco Inc. and Torii Pharmaceutical Co., Ltd., Tokyo, Japan) is used as an oral phosphate binder for the treatment of hyperphosphatemia in patients with chronic kidney disease (CKD) in Japan. Previous studies in Japanese patients with CKD have shown that the Hb concentration, serum iron concentration, transferrin saturation and serum ferritin concentration were increased and that transferrin iron-binding capacity (TIBC) was decreased undergoing FC treatment (dose adjusted between 1.5 and 6.0 g/day), suggesting that these changes were due to the utilization of iron provided by FC [7–9]. The most frequent adverse event (AE) was diarrhea, and the incidences of nausea and vomiting were very low in the FC groups [7–9]. In the United States, a similar ferric citrate product (Auryxia^®^; Akebia Therapeutics, Inc., Cambridge, MA, USA) has been approved as both a phosphate binder in patients with CKD undergoing dialysis and an iron-replacement agent in patients with CKD not undergoing dialysis [10]. Therefore, we expected that FC had potential to be an effective and well-tolerated oral iron preparation for the treatment of IDA.

We have already conducted two clinical trials (a clinical pharmacology study and a phase 2 dose–response study) in Japanese patients with IDA. In the clinical pharmacology study, we observed a similar increase in the serum iron concentration by administration of a single 500-mg dose of FC regardless of meals. Furthermore, in the phase 2 dose–response study, we confirmed that the change in the Hb concentration at the end of the treatment from baseline was significantly greater in the FC ≥ 250-mg/day dosage groups than in the placebo group after 7 weeks of treatment and increased in a dose-dependent manner from 250 to 1500 mg/day. A sodium ferrous citrate (SF) group (100 mg/day, 100 mg Fe/day) served as the reference group in this study, and the least-squares mean (LSM) change in the Hb concentration at the end of treatment in this group was 3.15 g/dL [95% confidence interval (CI) 2.78–3.52], which was comparable to that in patients taking FC at 500 mg/day (2.59 g/dL; 95% CI 2.16–3.01) and 1000 mg/day (3.33 g/dL; 95% CI 2.93–3.73).

The current study was a pivotal phase 3 clinical trial to evaluate the efficacy and safety of FC in patients with IDA compared with SF, which is widely used to treat IDA in Japan.

## Materials and methods

### Study design

This was a randomized, double-blind, active-controlled, multicenter non-inferiority study conducted at 50 centers in Japan. The study was conducted in accordance with the Declaration of Helsinki and Good Clinical Practice guidelines and was registered as JapicCTI-183999 in the JAPIC Clinical Trials Information. The study was conducted by Japan Tobacco, Inc. from July 2018 to March 2019. Study-related documents, including the protocol and informed consent forms, were approved by the institutional review boards. All patients provided written informed consent before any study procedures were initiated.

### Patients

Japanese outpatients aged ≥ 20 years who had been diagnosed with IDA were enrolled.

The inclusion criteria were as follows: (1) Hb concentration of ≥ 7.0 and < 11.0 g/dL at the screening visit (Scr Visit) and Week 0, respectively, with a difference of ≤ 1.0 g/dL between these two points and (2) serum ferritin concentration of < 12.0 ng/mL or TIBC of ≥ 360 μg/dL at the Scr Visit.

The main exclusion criteria were as follows: (1) anemia caused by conditions other than iron deficiency; (2) serum phosphorus concentration of < 2.5 mg/dL at the Scr Visit; (3) oral or intravenous iron administration within 4 weeks before the Scr Visit; (4) blood transfusion or the use of erythropoiesis-stimulating agents, protein anabolic hormones, testosterone enanthate, or mepitiostane within 12 weeks before the Scr Visit; (5) hepatic dysfunction or chronic hepatitis C; (6) gastrointestinal disease such as acute peptic ulcers, chronic ulcerative colitis, or regional enteritis; (7) paroxysmal nocturnal hemoglobinuria; and (8) inconstant treatment affecting the amount of bleeding for at least 8 weeks before Week 0.

The patients were randomly assigned to the FC-low group (FC at 500 mg/day, approximately 120 mg Fe/day), FC-high group (FC at 1000 mg/day, approximately 240 mg Fe/day), or SF group (SF at 100 mg/day) in a 1:1:1 ratio. Computer-generated randomization was performed with a dynamic allocation method, and the randomization was stratified by the Hb concentration at Week 0.

### Study treatment

**Fig. 1:**
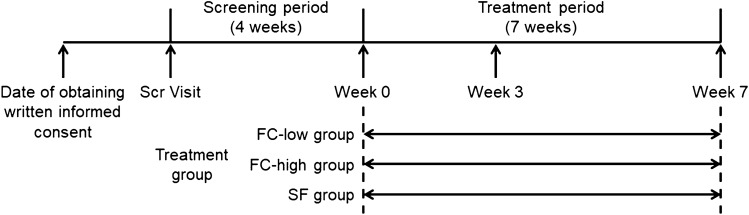
Outline of study schedule. *Scr Visit* screening visit, *FC-low group* ferric citrate hydrate at 500 mg/day (approximately 120 mg Fe/day), *FC-high group* ferric citrate hydrate at 1000 mg/day (approximately 240 mg Fe/day), *SF group* sodium ferrous citrate at 100 mg/day (100 mg Fe/day)

This study consisted of a 4-week screening period and a 7-week treatment period to administer the investigational products after randomization (Fig. 1). The patients in the FC-low and FC-high groups were administered two FC tablets once or twice daily immediately after a meal, respectively; whereas, the patients in the SF group were administered two SF tablets (containing 50 mg of iron per tablet) after a meal. The double-blind design required that the patients in the FC-low and FC-high groups received SF-matching placebo once daily, while the patients in the SF group received FC-matching placebo twice daily. The FC and SF tablets, including the matching placebos, were supplied by Japan Tobacco, Inc. and Sannova Co., Ltd. (Gunma, Japan), respectively.

Concomitant use of any iron preparations, blood transfusion, other drugs that may affect hematopoiesis, and drugs for the treatment of hyperphosphatemia were prohibited during the study. The dosage of drugs that apparently affect the amount of menstrual bleeding was kept constant, and surgical treatments for the bleeding were prohibited from the Scr Visit to Week 7 or the observation day at the time of discontinuation.

### Study population and study endpoints

Efficacy and safety analyses were performed on the modified intention-to-treat (mITT) population and the safety analysis population, respectively. Both populations comprised all randomized patients who were assessed for efficacy (mITT population) or safety (safety analysis population) at least once after receiving the study drugs.

The efficacy endpoints were defined as Hb, red blood cells (RBC), hematocrit, mean corpuscular volume (MCV), reticulocytes (erythrocyte-related parameters), iron, ferritin, TIBC, transferrin saturation (TSAT), hepcidin-25 and soluble transferrin receptor (sTfR) (iron-related parameters). The laboratory tests were performed at the central laboratory. The hepcidin-25 concentration was determined by Quantikine^®^ ELISA Human Hepcidin Immunoassay (R&D systems, Inc.), and sTfR concentration was measured with Access sTfR assay in the Access immunoassay system (Beckman Coulter Inc.).

The primary endpoint was the change in the Hb concentration from baseline to Week 7 in the mITT population. The secondary endpoints were the cumulative proportion of patients who achieved the target Hb concentration at Week 7, erythrocyte- and iron-related parameters and the cumulative proportion of patients who achieved an increase in the Hb concentration of ≥ 2.0 g/dL from baseline at Week 7. The target Hb concentration was set as ≥ 13.0 g/dL in male patients and ≥ 12.0 g/dL in female patients.

Safety assessments were based on symptoms, vital signs and laboratory test results in the safety analysis population. It was also important to precisely compare the incidence of gastrointestinal AEs such as nausea, vomiting and diarrhea between the FC and SF treatment groups; therefore, the investigators gathered information on these AEs by referring to patient-answered checklists and interviews.

### Statistical analysis

Based on the results of a phase 2 study, we assumed that the change in the Hb concentration from baseline (mean ± standard deviation) would be 2.60 ± 1.10 g/dL in the FC-low group and 3.20 ± 1.10 g/dL in the SF group, and we set the non-inferiority margin at − 1.00 g/dL. In this case, the required sample size was 160 patients per group to confirm that the lower limit of the 95% CI for the difference in the change in the Hb concentration between the FC-low group and SF group was over the non-inferiority margin with power of 90%.

The primary endpoint was analyzed using a mixed model for repeated measures with treatment, visit, and interaction between treatment and visit as fixed effects and the baseline Hb concentration as a covariate. When the non-inferiority of the FC-high group compared with the SF group was confirmed, we also tested the non-inferiority of the FC-low group versus the SF group with the fixed sequence procedure.

The cumulative proportion of patients who achieved the target Hb concentration was analyzed using a logistic regression model with multiple imputation including treatment as a fixed effect and the baseline Hb concentration as a covariate.

All AEs and adverse drug reactions (ADRs) were coded using MedDRA/J V.21.0. The incidence of nausea or vomiting was compared between each FC group and the SF group using Fisher’s exact test without multiplicity adjustments.

All statistical analyses were conducted using SAS^®^ 9.4 (SAS Institute Inc., Cary, NC, USA).

## Results

### Patients

**Fig. 2:**
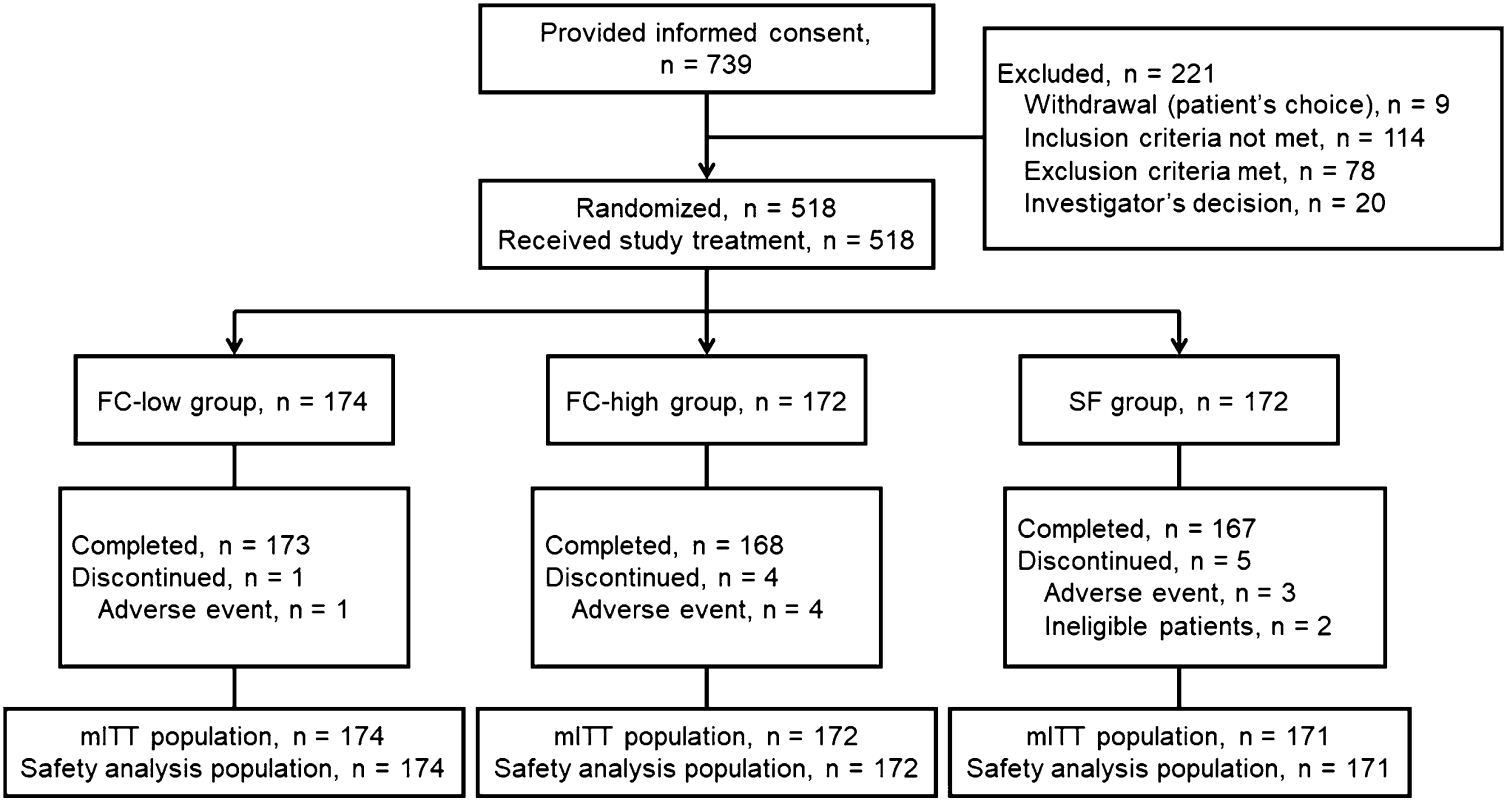
Patient disposition. *FC-low group* ferric citrate hydrate at 500 mg/day, *FC-high group* ferric citrate hydrate at 1000 mg/day, *SF group* sodium ferrous citrate at 100 mg/day, *mITT* modified intention-to-treat

A total of 739 patients consented to participate in this study, and 518 eligible patients were randomized to the FC-low group (*n* = 174), FC-high group (*n* = 172) and SF group (*n* = 172) (Fig. 2). All randomized patients received the study treatment, and 508 of these patients completed the study. One patient in the SF group was excluded from all analysis populations because of a serious protocol violation (deliberate double registration). Finally, the mITT population and safety analysis population included 174 patients in the FC-low group, 172 patients in the FC-high group and 171 patients in the SF group. There were no apparent differences in patient demographics or baseline values among the treatment groups. Almost all enrolled patients were premenopausal female and the mean age of the overall study population was 40.7 years (Table 1). Drug adherence was 96.8% in the FC-low group, 96.4% in the FC-high group and 95.1% in the SF group.

**Table 1:** Patient demographics (mITT population)

Characteristics	FC-low (*n* = 174)	FC-high (*n* = 172)	SF (*n* = 171)	Total (*n* = 517)
Age, years	41.1 (7.4)	41.1 (7.3)	40.1 (7.2)	40.7 (7.3)
Sex				
Male	1 (0.6)	0 (0.0)	0 (0.0)	1 (0.2)
Female	173 (99.4)	172 (100.0)	171 (100.0)	516 (99.8)
Menopausal status (female only)				
Pre-menopause	172 (99.4)	168 (97.7)	171 (100.0)	511 (99.0)
Post-menopause	1 (0.6)	4 (2.3)	0 (0.0)	5 (1.0)
Primary disease underlying IDA				
Uterine myoma	50 (28.7)	45 (26.2)	41 (24.0)	136 (26.3)
Adenomyosis uteri	16 (9.2)	18 (10.5)	26 (15.2)	60 (11.6)
Endometriosis	6 (3.4)	6 (3.5)	10 (5.8)	22 (4.3)
Others	58 (33.3)	63 (36.6)	65 (38.0)	186 (36.0)
Unknown	50 (28.7)	50 (29.1)	43 (25.1)	143 (27.7)
Previous iron treatment experience				
No	66 (37.9)	70 (40.7)	63 (36.8)	199 (38.5)
Yes	108 (62.1)	102 (59.3)	108 (63.2)	318 (61.5)

Data are presented as mean (standard deviation) or *n* (%)

*mITT* modified intention-to-treat, *FC-low group* ferric citrate hydrate at 500 mg/day, *FC-high group* ferric citrate hydrate at 1000 mg/day, *SF group* sodium ferrous citrate at 100 mg/day, *IDA* iron deficiency anemia

### Efficacy

#### Primary endpoint

The LSM change in the Hb concentration at Week 7 from baseline was 3.29 g/dL in the FC-high group and 3.11 g/dL in the SF group, and the difference between these groups was 0.18 g/dL (95% CI − 0.01 to 0.37) (Table 2). The lower limit of the 95% CI of the difference between these groups was higher than the predefined non-inferiority margin of − 1.00 g/dL; hence, non-inferiority of FC at 1000 mg/day relative to SF at 100 mg/day was confirmed.

**Table 2:** Non-inferiority assessment of Hb changes from baseline at Week 7 between FC groups and SF group (mITT population)

Visit	Step	Group	*n*	Change in Hb concentration from baseline		Difference vs. SF group	
				LSM	95% CI	LSM	95% CI
Week 7	Step 1	FC-high	171	3.29	3.15–3.42	0.18	− 0.01 to 0.37
		SF	169	3.11	2.97–3.24	–	–
	Step 2	FC-low	173	2.75	2.62–2.88	− 0.36	− 0.55 to − 0.18
		SF	169	3.11	2.98–3.24	–	–

*Hb* hemoglobin, *mITT* modified intention-to-treat, *LSM* least-squares mean, *CI* confidence interval, *FC-low group* ferric citrate hydrate at 500 mg/day, *FC-high group* ferric citrate hydrate at 1000 mg/day, *SF group* sodium ferrous citrate at 100 mg/day

The LSM change in the Hb concentration at Week 7 from baseline was 2.75 g/dL in the FC-low group and 3.11 g/dL in the SF group, and the difference between these groups was − 0.36 g/dL (95% CI − 0.55 to − 0.18) (Table 2). This result showed non-inferiority of FC at 500 mg/day to SF at 100 mg/day, as with FC at 1000 mg/day.

#### Secondary endpoints

**Fig. 3:**
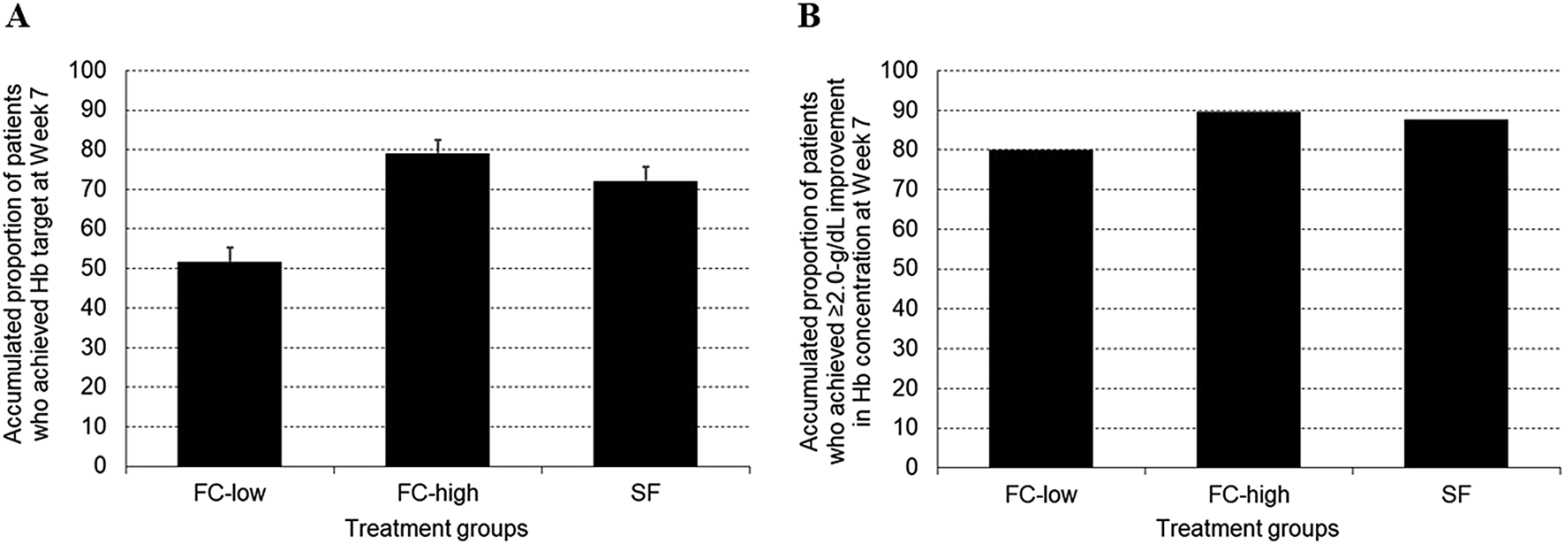
**a** Accumulated proportion of patients who achieved the Hb target concentration at Week 7 (mITT population). **b** Accumulated proportion of patients who achieved ≥ 2.0 g/dL improvement in Hb concentration at Week 7 (mITT population). *Hb* hemoglobin, *FC-low group* ferric citrate hydrate at 500 mg/day, *FC-high group* ferric citrate hydrate at 1000 mg/day, *SF group* sodium ferrous citrate at 100 mg/day

The adjusted proportion of patients who achieved the Hb target at Week 7 was 51.5% in the FC-low group, 79.1% in the FC-high group and 72.1% in the SF group (Fig. 3a). The odds ratio (vs. SF group) obtained from the logistic regression analysis was 0.46 (95% CI 0.35–0.61) in the FC-low group and 1.80 (95% CI 1.33–2.43) in the FC-high group (Table 3). The logistic regression analysis suggested that the cumulative proportion of patients who achieved the target Hb concentration at Week 7 was the highest in those treated with FC at 1000 mg/day, followed by SF at 100 mg/day and FC at 500 mg/day.

**Table 3:** Logistic regression analysis of accumulated proportion of patients who achieved target hemoglobin concentration at Week 7 (mITT population)

Visit	Parameter	FC-low (*n* = 174) vs. SF (*n* = 171)	FC-high (*n* = 172) vs. SF (*n* = 171)
Week 7	Odds ratio	0.46	1.80
	95% CI	0.35–0.61	1.33–2.43

*mITT* modified intention-to-treat, *CI* confidence interval, *FC-low group* ferric citrate hydrate at 500 mg/day, *FC-high group* ferric citrate hydrate at 1000 mg/day, *SF group* sodium ferrous citrate at 100 mg/day

The proportion of patients who achieved a ≥ 2.0 g/dL improvement in the Hb concentration at Week 7 was 79.9% in the FC-low group, 89.5% in the FC-high group and 87.7% in the SF group (Fig. 3b).

The time course of erythrocyte- and iron-related parameters was similar in all groups (Table 4). The increases in the Hb concentration, hematocrit level, RBC count and MCV level persisted throughout the treatment period. The number of reticulocytes increased at Week 3 and decreased until Week 7. These results indicate that the anemic state was improved by the treatment with FC as well as SF. Moreover, the serum iron concentration, ferritin concentration, TSAT level and hepcidin-25 concentration increased, and the TIBC level and sTfR concentration decreased at Week 7 in all groups, demonstrating that FC led to recovery of the iron-deficiency state, as did SF.

**Table 4:** Time course of erythrocyte/iron-related parameters (mITT population) and phosphorus (safety analysis population)

Parameters	FC-low	FC-high	SF
Hemoglobin, g/dL			
Baseline	9.25 ± 0.92 (*n* = 174)	9.24 ± 0.89 (*n* = 172)	9.24 ± 0.87 (*n* = 171)
Week 3	10.76 ± 0.84 (*n* = 173)	11.32 ± 0.87 (*n* = 170)	11.11 ± 0.82 (*n* = 167)
Week 7	11.99 ± 0.90 (*n* = 173)	12.55 ± 0.95 (*n* = 168)	12.35 ± 0.85 (*n* = 167)
RBC, 10^4^/µL			
Baseline	416.5 ± 39.3 (*n* = 174)	412.1 ± 34.3 (*n* = 172)	409.7 ± 32.9 (*n* = 171)
Week 3	451.3 ± 39.8 (*n* = 173)	454.9 ± 39.4 (*n* = 170)	450.9 ± 39.0 (*n* = 167)
Week 7	460.8 ± 42.8 (*n* = 173)	464.0 ± 38.1 (*n* = 168)	460.2 ± 37.2 (*n* = 167)
Hct, %			
Baseline	29.54 ± 2.46 (*n* = 174)	29.49 ± 2.42 (*n* = 172)	29.39 ± 2.32 (*n* = 171)
Week 3	33.98 ± 2.33 (*n* = 173)	35.49 ± 2.63 (*n* = 170)	34.85 ± 2.36 (*n* = 167)
Week 7	37.00 ± 2.71 (*n* = 173)	38.34 ± 2.73 (*n* = 168)	37.85 ± 2.49 (*n* = 167)
MCV, fL			
Baseline	71.3 ± 6.3 (*n* = 174)	71.8 ± 5.6 (*n* = 172)	72.0 ± 5.8 (*n* = 171)
Week 3	75.6 ± 5.6 (*n* = 173)	78.2 ± 4.3 (*n* = 170)	77.6 ± 4.8 (*n* = 167)
Week 7	80.6 ± 5.3 (*n* = 173)	82.8 ± 4.1 (*n* = 168)	82.4 ± 4.2 (*n* = 167)
Retic, 10^4^/µL			
Baseline	5.94 ± 2.03 (*n* = 174)	5.62 ± 2.13 (*n* = 172)	5.27 ± 1.91 (*n* = 171)
Week 3	9.14 ± 3.96 (*n* = 173)	9.51 ± 3.51 (*n* = 170)	8.97 ± 3.17 (*n* = 167)
Week 7	6.58 ± 2.72 (*n* = 173)	6.12 ± 2.72 (*n* = 168)	5.71 ± 2.48 (*n* = 167)
Iron, µg/dL			
Baseline	19.9 ± 20.7 (*n* = 174)	17.9 ± 9.1 (*n* = 172)	16.9 ± 7.4 (*n* = 171)
Week 3	41.3 ± 25.2 (*n* = 173)	76.1 ± 51.4 (*n* = 169)	51.8 ± 24.1 (*n* = 167)
Week 7	53.5 ± 31.4 (*n* = 173)	79.1 ± 44.0 (*n* = 167)	65.0 ± 40.9 (*n* = 167)
Ferritin, ng/mL			
Baseline	4.45 ± 3.98 (*n* = 174)	4.22 ± 2.48 (*n* = 172)	4.29 ± 2.96 (*n* = 171)
Week 3	20.46 ± 12.38 (*n* = 173)	30.66 ± 17.24 (*n* = 169)	35.05 ± 17.30 (*n* = 167)
Week 7	20.86 ± 11.69 (*n* = 173)	25.02 ± 12.59 (*n* = 168)	28.97 ± 21.10 (*n* = 167)
TIBC, g/dL			
Baseline	436.6 ± 48.6 (*n* = 174)	428.7 ± 44.0 (*n* = 172)	429.8 ± 40.5 (*n* = 171)
Week 3	404.6 ± 48.1 (*n* = 173)	365.1 ± 41.7 (*n* = 169)	382.6 ± 41.5 (*n* = 167)
Week 7	372.7 ± 47.1 (*n* = 173)	340.5 ± 45.1 (*n* = 167)	346.8 ± 38.7 (*n* = 167)
TSAT, %			
Baseline	4.6 ± 4.7 (*n* = 174)	4.2 ± 2.1 (*n* = 172)	3.9 ± 1.8 (*n* = 171)
Week 3	10.4 ± 6.6 (*n* = 173)	20.8 ± 13.4 (*n* = 169)	13.8 ± 6.7 (*n* = 167)
Week 7	14.6 ± 8.8 (*n* = 173)	23.4 ± 12.0 (*n* = 167)	19.0 ± 12.1 (*n* = 167)
Hepcidin-25, ng/L			
Baseline	208.7 ± 301.4 (*n* = 174)	213.1 ± 317.5 (*n* = 172)	364.3 ± 1362.1 (*n* = 171)
Week 7	4166.1 ± 7987.1 (*n* = 173)	9173.2 ± 7997.5 (*n* = 168)	7439.1 ± 9891.3 (*n* = 167)
sTfR, nmol/L			
Baseline	44.75 ± 14.17 (*n* = 174)	46.36 ± 16.31 (*n* = 172)	44.84 ± 18.29 (*n* = 171)
Week 7	24.52 ± 7.82 (*n* = 173)	21.82 ± 6.95 (*n* = 168)	22.48 ± 6.98 (*n* = 167)
Phosphorus, mg/dL			
Baseline	3.50 ± 0.45 (*n* = 174)	3.55 ± 0.50 (*n* = 172)	3.57 ± 0.46 (*n* = 171)
Week 3	3.59 ± 0.47 (*n* = 174)	3.61 ± 0.52 (*n* = 170)	3.59 ± 0.46 (*n* = 170)
Week 7	3.62 ± 0.50 (*n* = 173)	3.65 ± 0.51 (*n* = 168)	3.62 ± 0.49 (*n* = 168)

Data are presented as mean ± standard deviation

*mITT* modified intention-to-treat, *RBC* red blood cells, *Hct* hematocrit, *MCV* mean corpuscular volume, *Retic* reticulocytes, *TIBC* total iron-binding capacity, *TSAT* transferrin saturation, *sTfR* soluble transferrin receptor, *FC-low group* ferric citrate hydrate at 500 mg/day, *FC-high group* ferric citrate hydrate at 1000 mg/day, *SF group* sodium ferrous citrate at 100 mg/day

### Safety and tolerability

No deaths and no severe AEs were reported during the study.

AEs were reported in 85 (48.9%) patients in the FC-low group, 84 (48.8%) in the FC-high group and 109 (63.7%) in the SF group. Likewise, ADRs were reported in 52 (29.9%) patients in the FC-low group, 50 (29.1%) in the FC-high group and 72 (42.1%) in the SF group. The incidences of AEs and ADRs in each FC treatment group were lower than those in the SF group. One serious AE occurred in each group, and one headache in the FC-low group was considered to be related to the study treatment. Most of the AEs and ADRs were considered mild.

The most common AEs by system organ classes were gastrointestinal disorders, which occurred in 61 (35.1%) patients in the FC-low group, 60 (34.9%) in the FC-high group and 83 (48.5%) in the SF group (Table 5). With the exception of the above-mentioned serious AEs, almost all AEs leading to early discontinuation of the study treatments were gastrointestinal-related symptoms (excluding one case of eczema in the FC-high group).

**Table 5:** Summary of common adverse events (safety analysis population)

	FC-low (*n* = 174)	FC-high (*n* = 172)	SF (*n* = 171)
Gastrointestinal disorders	61 (35.1)	60 (34.9)	83 (48.5)
Constipation	8 (4.6)	6 (3.5)	0 (0.0)
Diarrhea	42 (24.1)	48 (27.9)	40 (23.4)
Nausea	27 (15.5)	18 (10.5)	56 (32.7)
Vomiting	9 (5.2)	2 (1.2)	26 (15.2)
Infections and infestations	27 (15.5)	19 (11.0)	29 (17.0)
Nasopharyngitis	16 (9.2)	12 (7.0)	19 (11.1)
Investigations	2 (1.1)	2 (1.2)	12 (7.0)
Increase in γ-GTP	0 (0.0)	0 (0.0)	6 (3.5)

Adverse events occurring in ≥ 2.0% of patients in either treatment group are listed. Data are presented as *n* (%)

*FC-low group* ferric citrate hydrate at 500 mg/day, *FC-high group* ferric citrate hydrate at 1000 mg/day, *SF group* sodium ferrous citrate at 100 mg/day, *γ-GTP* gamma-glutamyl transpeptidase

**Fig. 4:**
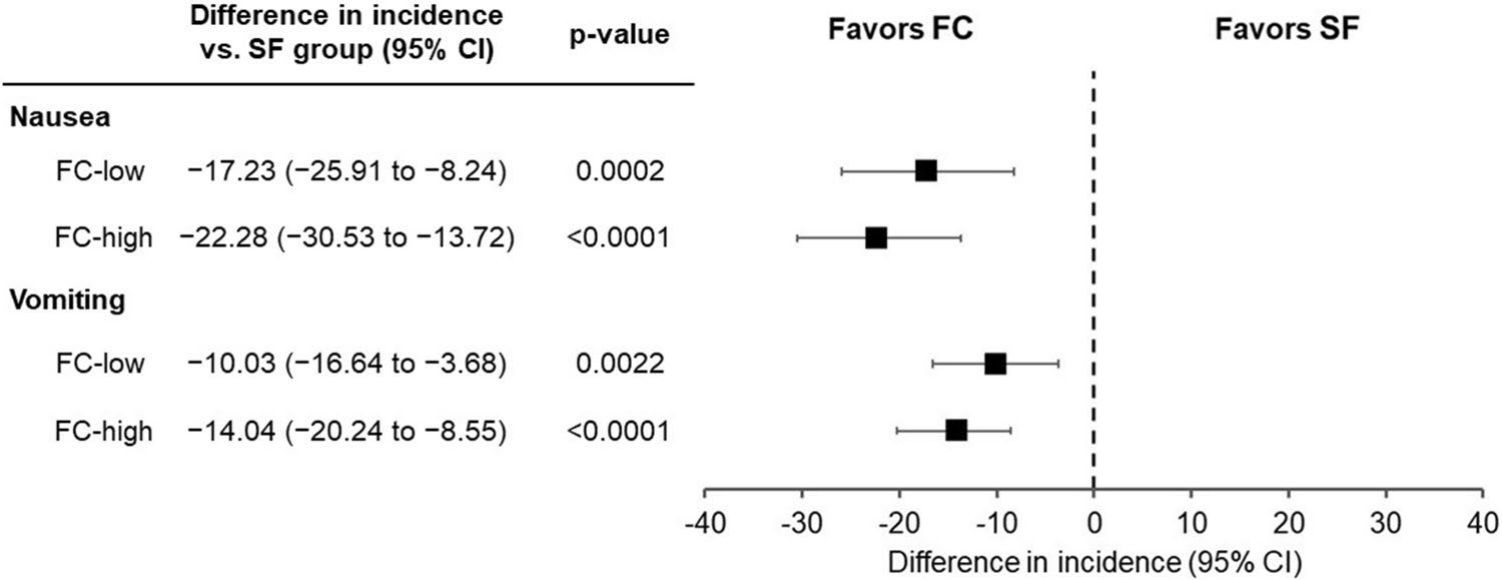
Differences in incidence of nausea and vomiting as adverse events between FC and SF groups (safety analysis population). *FC-low group* ferric citrate hydrate at 500 mg/day, *FC-high group* ferric citrate hydrate at 1000 mg/day, *SF group* sodium ferrous citrate at 100 mg/day, *CI* confidence interval

The most frequently reported AE in the FC treatment groups was diarrhea (Table 5). The incidence of diarrhea was similar among the three groups. In contrast, the most frequently reported AE in the SF group was nausea. The incidence of nausea was significantly lower in the FC treatment groups than in the SF group (Fig. 4). Moreover, the incidence of vomiting was also significantly lower in the FC treatment groups than in the SF group (Fig. 4). The same results were found for ADRs (Supplementary Table 1; Supplementary Fig. 1). These gastrointestinal AEs were observed early in the treatment periods.

No clinically relevant changes in vital signs or laboratory parameters were found throughout the treatment period in any of the three treatment groups. In particular, no major changes in the serum phosphorous concentration were found in any of the groups (Table 4). There were no new concerns in the FC-high group compared with the FC-low group.

## Discussion

The oral iron preparations available for adult patients with IDA in Japan are SF, dried iron sulfate and ferrous fumarate. Oral iron administration increases the number of reticulocytes within several days, and the Hb concentration returns to the normal range after 6–8 weeks of treatment [11]. This suggests that the currently available oral iron therapies have sufficient efficacy to treat patients with IDA. However, the existing oral iron preparations have unpleasant gastrointestinal side effects such as nausea, vomiting, abdominal pain and constipation [1, 12]. Patients’ experience with these symptoms is a reason why patients discontinue the appropriate treatment for IDA [5, 6]. Although the administration of intravenous iron preparations can replenish body iron stores very quickly, there are problems associated with the highly invasive injection and concerns about side effects such as allergic reactions and hypersensitivity [13]. Therefore, it is worth developing FC as an oral iron preparation because this agent has a fewer gastrointestinal side effects and improves the treatment adherence compared with the existing oral iron preparations.

The results of the primary endpoint analysis showed that both FC at 500 and 1000 mg/day, given for 7 weeks, was non-inferior to SF at 100 mg/day with respect to the mean change in the Hb concentration. This finding suggests that both the dosages of FC have good efficacy in the treatment of IDA, similar to the existing oral iron preparations. In addition, the cumulative proportion of patients who achieved the target Hb concentration at Week 7 was higher in the FC-high group than the FC-low group, suggesting that FC at 1000 mg/day has a stronger effect in the improvement of anemia than FC at 500 mg/day. However, approximately 80% of the patients in the FC-low group achieved a 2.0-g/dL increase in the Hb concentration at Week 7. Further analysis is needed, but it is possible that FC at 500 mg/day is sufficient to improve the anemic state in patients with mild anemia whose Hb concentration is around 10.0 g/dL; this dosage of FC may also improve patients’ adherence because of its easy dose regimen (two tablets once daily).

Safety assessments have shown good tolerability of FC in the treatment of IDA. The incidence of side effects of oral iron preparations generally increases with higher doses [14]; however, there were no major differences in the incidences of AEs and ADRs between the FC-low group and FC-high group in the present study.

Moreover, the incidence of nausea and vomiting in both FC treatment groups was 3–10 times lower than that in the SF group, and there were significant differences between the FC treatment groups and the SF group. These results indicate that FC has potential to be an effective oral iron preparation with a low risk of gastrointestinal-related side effects. Although the signal pathways inducing nausea and vomiting remain unclear, nausea and vomiting are reportedly caused by the stimulation of a vomiting center in the medulla oblongata through serotonin-3 receptors [15]. Chemotherapeutic agents generate free radicals, leading to the release of serotonin from enterochromaffin cells in the duodenal mucosa and inducing nausea and vomiting [16, 17]. Based on the reports that ferrous iron also generates free radicals [18], it is assumed that oral iron preparations including ferrous iron cause nausea and vomiting as side effects through mechanisms similar to those of chemotherapeutic agents. In fact, all oral iron preparations available for adult patients with IDA in Japan contain ferrous iron. We consider that FC is reduced to ferrous iron (Fe^2+^) by duodenal cytochrome B (Dcytb), then absorbed in the duodenum and proximal jejunum. Based on that, administration of FC would result in a small amount of total ferrous iron in the duodenal lumen, compared to administration of SF composed of ferrous iron (Fe^2+^). Therefore, FC might generate fewer free radicals than ferrous iron preparations, leading to lower incidences of nausea and vomiting during treatment.

The incidence of diarrhea as an AE or ADR was not significantly different among the treatment groups in this study. Free radicals generated by ferrous iron also reportedly induce mucosal damage in the intestine, leading to diarrhea [10, 19, 20]. As shown above, FC is reduced to ferrous iron by Dcytb over time. Hence, it is possible that the total amount of ferrous iron in the lower intestine by administration of FC is similar level as that by administration of SF. Therefore, both FC and SF might cause diarrhea during treatment.

FC is used as a phosphate binder to control the serum phosphorus concentration in adult patients with CKD. However, FC treatment resulted in no major changes in the serum phosphorus concentration in patients with IDA in the present study. One of the main reasons for this might be the difference in treatment dosages. The FC treatment dosage in this study was 500 mg once or twice daily, which is lower than the approved dosage (500–2000 mg three times daily) to treat hyperphosphatemia in patients with CKD in Japan. Furthermore, in other research, high-dose FC treatment decreased the serum phosphorus concentration; whereas, low-dose FC treatment had no effects on the phosphorus concentration in healthy rats with normal renal function [21, 22]. Non-clinical studies have also shown that low-dose FC treatment does not significantly change the concentrations of intact fibrosis growth factor 23 (iFGF23) and intact parathyroid hormone (iPTH), which play important roles in the metabolism of phosphorus, in healthy rats [21, 22]. Taking these results into consideration, we suggest that FC at 500 or 1000 mg/day will not adversely affect phosphorus metabolism during treatment in patients with IDA who have normal kidney function. The present study also showed that SF treatment had no effects on the serum phosphorus concentration in patients with IDA. However, some reports have indicated that saccharated ferric oxide and ferric carboxymaltose, which are intravenous iron preparations available in Japan, sometimes cause hypophosphatemia in patients with IDA [23, 24]. Therefore, overall, we presume that the lack of clinically significant effects on phosphorus metabolism is a major advantage of oral iron preparations, including FC, over intravenous iron preparations in patients with IDA who have normal kidney function.

In conclusion, we have demonstrated that FC at both 500 and 1000 mg/day is non-inferior to SF at 100 mg/day in terms of efficacy and has good tolerability in patients with IDA. Furthermore, FC was associated with significantly lower incidences of nausea and vomiting during treatment than was SF. These results indicate that FC is an oral iron preparation with sufficient efficacy for the treatment of IDA and low risks of nausea and vomiting. FC has high gastrointestinal safety and may be a potent alternative to existing oral iron preparations for patients with IDA.

### Supplementary Information

Below is the link to the electronic supplementary material.

Supplementary file 1 (DOCX 19 KB)

Supplementary file2 Differences in incidence of nausea and vomiting as adverse drug reactions between FC and SF groups (safety analysis population). FC-low group, ferric citrate hydrate at 500 mg/day; FC-high group, ferric citrate hydrate at 1000 mg/day; SF group, sodium ferrous citrate at 100 mg/day; CI, confidence interval (JPG 62 KB)
Supplementary Fig. 1 Differences in incidence of nausea and vomiting as adverse drug reactions between FC and SF groups (safety analysis population). FC-low group, ferric citrate hydrate at 500 mg/day; FC-high group, ferric citrate hydrate at 1000 mg/day; SF group, sodium ferrous citrate at 100 mg/day; CI, confidence interval
