# Correction: Efficacy and safety of ferric citrate hydrate compared with sodium ferrous citrate in Japanese patients with iron deficiency anemia: a randomized, double-blind, phase 3 non-inferiority study

**DOI:** 10.1007/s12185-024-03739-7

**Published:** 2024-03-06

**Authors:** Norio Komatsu, Kojo Arita, Hironori Mitsui, Takanori Nemoto, Koji Hanaki

**Affiliations:** 1 Department of Hematology, Juntendo University School of Medicine, 2-1-1 Hongo, Bunkyo-ku, Tokyo, 113-8421 Japan; 2 Pharmaceutical Division, Japan Tobacco Inc., 3-4-1 Nihonbashi-Honcho, Chuo-ku, Tokyo, 103-0023 Japan

**Correction: International Journal of Hematology (2021) 114:8–17** 10.1007/s12185-021-03123-9

The article “Efficacy and safety of ferric citrate hydrate compared with sodium ferrous citrate in Japanese patients with iron deficiency anemia: a randomized, double-blind, phase 3 non-inferiority study”, written by Norio Komatsu, Kojo Arita, Hironori Mitsui, Takanori Nemoto, Koji Hanaki, was originally published electronically on the publisher’s internet portal on 15 March 2021 without open access. With the author(s)’ decision to opt for Open Choice the copyright of the article changed on 19 February 2024 to © The Author(s) 2024 and the article is forthwith distributed under a Creative Commons Attribution 4.0 International License, which permits use, sharing, adaptation, distribution and reproduction in any medium or format, as long as you give appropriate credit to the original author(s) and the source, provide a link to the Creative Commons licence, and indicate if changes were made. The images or other third party material in this article are included in the article's Creative Commons licence, unless indicated otherwise in a credit line to the material. If material is not included in the article's Creative Commons licence and your intended use is not permitted by statutory regulation or exceeds the permitted use, you will need to obtain permission directly from the copyright holder. To view a copy of this licence, visit http://creativecommons.org/licenses/by/4.0/. The original article has been corrected.

