# Supplementary material for: Efficacy and safety of ferric citrate hydrate compared with sodium ferrous citrate in Japanese patients with iron deficiency anemia: a randomized, double-blind, phase 3 non-inferiority study
Source: Int J Hematol. 2021 Mar 15;114(1):8–17. doi: 10.1007/s12185-021-03123-9 (PMC10917848; doi:10.1007/s12185-021-03123-9)
Supplement: Supplementary file 1 — (DOCX 19 KB) [file 12185_2021_3123_MOESM1_ESM.docx]

**Supplementary Table 1. Summary of common adverse drug reactions (safety analysis population)***

|  | **FC-low (n = 174)** | **FC-high (n = 172)** | **SF (n = 171)** |
| --- | --- | --- | --- |
| Gastrointestinal disorders | 51 (29.3) | 46 (26.7) | 66 (38.6) |
| Constipation | 5 (2.9) | 6 (3.5) | 0 (0.0) |
| Diarrhea | 31 (17.8) | 36 (20.9) | 30 (17.5) |
| Nausea | 21 (12.1) | 13 (7.6) | 49 (28.7) |
| Vomiting | 5 (2.9) | 0 (0.0) | 21 (12.3) |
| Investigations | 1 (0.6) | 0 (0.0) | 6 (3.5) |
| Increase in γ-GTP | 0 (0.0) | 0 (0.0) | 4 (2.3) |

Data are presented as n (%).

FC-low group, ferric citrate hydrate at 500 mg/day; FC-high group, ferric citrate hydrate at 1000 mg/day; SF group, sodium ferrous citrate at 100 mg/day; γ-GTP, gamma-glutamyl transpeptidase

*Adverse drug reactions occurring in ≥2.0% of patients in either treatment group are listed.
