# Supplementary figures and images for: Efficacy and safety of ferric citrate hydrate compared with sodium ferrous citrate in Japanese patients with iron deficiency anemia: a randomized, double-blind, phase 3 non-inferiority study
Source: Int J Hematol. 2021 Mar 15;114(1):8–17. doi: 10.1007/s12185-021-03123-9 (PMC10917848; doi:10.1007/s12185-021-03123-9)

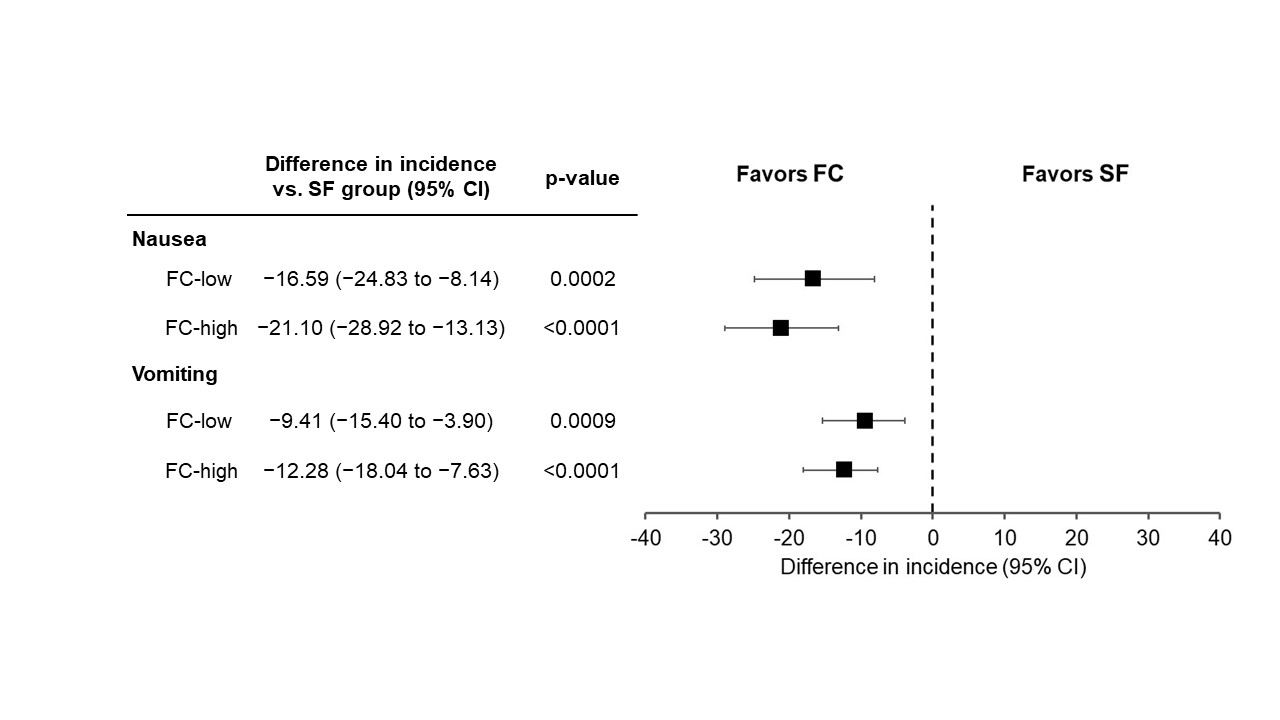

Supplement: Supplementary file 2 — Supplementary file2 Differences in incidence of nausea and vomiting as adverse drug reactions between FC and SF groups (safety analysis population). FC-low group, ferric citrate hydrate at 500 mg/day; FC-high group, ferric citrate hydrate at 1000 mg/day; SF group, sodium ferrous citrate at 100 mg/day; CI, confidence interval (JPG 62 KB) Supplementary Fig. 1 Differences in incidence of nausea and vomiting as adverse drug reactions between FC and SF groups (safety analysis population). FC-low group, ferric citrate hydrate at 500 mg/day; FC-high group, ferric citrate hydrate at 1000 mg/day; SF group, sodium ferrous citrate at 100 mg/day; CI, confidence interval [file 12185_2021_3123_MOESM2_ESM.jpg]
